# Compressive Strength Prediction of PVA Fiber-Reinforced Cementitious Composites Containing Nano-SiO_2_ Using BP Neural Network

**DOI:** 10.3390/ma13030521

**Published:** 2020-01-22

**Authors:** Ting-Yu Liu, Peng Zhang, Juan Wang, Yi-Feng Ling

**Affiliations:** 1 School of Water Conservancy Engineering, Zhengzhou University, Zhengzhou 450001, China; my19990312@163.com (T.-Y.L.); wangjuan@zzu.edu.cn (J.W.); 2 National Concrete Pavement Technology Center, Institute for Transportation, Ames, IA 50010, USA; yling@iastate.edu

**Keywords:** BP neural network, cementitious composite, nano-SiO_2_, PVA fiber, genetic algorithm

## Abstract

In this study, a method to optimize the mixing proportion of polyvinyl alcohol (PVA) fiber-reinforced cementitious composites and improve its compressive strength based on the Levenberg-Marquardt backpropagation (BP) neural network algorithm and genetic algorithm is proposed by adopting a three-layer neural network (TLNN) as a model and the genetic algorithm as an optimization tool. A TLNN was established to implement the complicated nonlinear relationship between the input (factors affecting the compressive strength of cementitious composite) and output (compressive strength). An orthogonal experiment was conducted to optimize the parameters of the BP neural network. Subsequently, the optimal BP neural network model was obtained. The genetic algorithm was used to obtain the optimum mix proportion of the cementitious composite. The optimization results were predicted by the trained neural network and verified. Mathematical calculations indicated that the BP neural network can precisely and practically demonstrate the nonlinear relationship between the cementitious composite and its mixture proportion and predict the compressive strength. The optimal mixing proportion of the PVA fiber-reinforced cementitious composites containing nano-SiO_2_ was obtained. The results indicate that the method used in this study can effectively predict and optimize the compressive strength of PVA fiber-reinforced cementitious composites containing nano-SiO_2_.

## 1. Introduction

Concrete is a widely used building material in engineering constructions [1]. With the large-scale construction of long-span bridges, super-high-rise buildings, high-grade highways, large-scale water conservancy facilities, and cross harbor tunnels, concrete materials are endowed with higher expectations [2]. More problems have been caused by traditional concrete materials, such as the crack propagation inside concrete materials and the lack of durability [1]. Therefore, it is crucial to optimize the mix proportion and improve the compressive strength of concrete [3]. Recently, researchers have added nanofiber additives in the concrete mixing process to optimize the performance of concrete.

Cementitious materials are becoming increasingly important for the future of the automated building industry [4]. Related research results indicated that cementitious materials work against environmental pollution by minimizing the emission of CO_2_, other pollutant gases and waste dust, exhibit important feasibility and application prospects, and may become an appropriate substitute for traditional cement mortar in the future [5]. Some researchers have produced some new materials to replace the traditional cement totally or in part, such as concrete incorporating ferronickel slag (FNS) as a replacement of natural sand [6] and engineered cementitious composite layered reinforced concrete beams [7]. Simultaneously, descriptions of fibers in cementitious composites containing polydispersed hollow and core–shell microparticles [8], waste recycled hollow glass microspheres [9], multiwalled carbon nanotubes [10], nano reservoir silts [11], SiO_2_ nanoparticles [12], palm oil fuel ash [13], inclined steel fiber [14], cellulose nanocrystals [15], cobalt ferrite and nanoparticles [16] are abundant. Polyvinyl alcohol (PVA) fiber-reinforced engineering cementitious composite is a kind of new high-performance cementitious material which exhibits the features of strain hardening, multiple-cracking high durability [17], and narrow crack width [18,19,20]. Additionally, it exhibits the characteristics of multislit cracking and strain hardening [21] and possesses a broad application prospect [22]. In recent years, PVA fiber-reinforced engineering cementitious composite has been extensively studied [23]. Li and Gao discussed the multiple effects of the fluidity, microstructure, and bending performance of cementitious composites with high-toughness reinforced by nano-SiO_2_ and hybrid fiber [24]. The results demonstrated that composites incorporated with 1.4% steel fibers and 2.5% PVA fibers exhibited good flexural performance. Qiu and Lim conducted an experimental study on the fatigue strength degradation of micro-PVA fiber in a cement matrix [25]. They discovered that the fiber embedded in the cement matrix reduced the in-situ strength of the fiber, and changed the fatigue properties of the fiber. Ranjbarian and Mechtcherine established a pre pull-out locking model for PVA microfibers embedded in a cementitious matrix [26].

Owing to the high nonlinearity and strong generalization ability of the neural network model, it is extensively used in the classification and prediction of complicated models. Recently, neural networks have been widely applied to the research and prediction of concrete material properties to study the nonlinear and complex relationships between concrete material properties and mix proportion. A large number of studies indicated that the nonlinear mapping relationship constructed using neural networks could deliver the performance of concrete materials, and neural network could be used to optimize the mix proportion of concrete materials. Tanja and Ivana processed a database compiled based on their experimental results of recycled brick aggregate concrete using neural network to obtain a reliable prediction, and they proposed an optimized quantitative model for proportioning concrete mixtures [27]. Haissam and Sudhir developed simple multilayer perceptron structure of Artificial Neural Network models using Marshall mix design data, and the models were called by a non-linear constrained genetic algorithm to optimize the asphalt mix, so as to achieve the prediction and optimization of asphalt mixture composition [28]. On the basis of a time-series model, Wang created an artificial neural network model of data mining to access the influence of cement curing stage on pozzolanic activity [29] and subsequently predicted the pozzolanic activity. Based on a series of tests, Ji and Lin established a prediction model of concrete strength and slump based on an artificial neural network [30]. Through the reverse derivation of the two prediction models, the calculation models to obtain the equivalent ratio of cement to water and the average paste thickness were established. The concrete designed using this algorithm had small cement and water content, more excellent durability, and higher economic and ecological benefits. Qi and Fourier used the neural network and particle swarm optimization algorithm to predict the unconfined compressive strength of cement paste filling [31], and the results indicated that the optimal artificial neural network model was highly accurate for the prediction of cemented paste backfill strength. Jian and Roy investigated the debonding behavior of high-performance fiber concrete and traditional concrete under a direct shear load and established a robust machine learning model to calculate the shear debonding strength of the concrete with influence parameters [32], which should corroborate the validity of the model in describing the debonding response of the concrete.

With the development of deep research on nanoparticles and the gradual reduction in manufacturing costs for nanoparticles [33], nanoparticles have been gradually extended to the application of civil engineering owing to their characteristic nano effects [34]. Simultaneously, fiber-reinforced composites are also widely used in the construction and building industry, such as plastic fibers as the only reinforcement for flat suspended slabs [35] and polyolefin fiber-reinforced concrete for infrastructure applications [36,37]. However, currently, systematic studies on PVA fiber-reinforced cementitious composites containing nano-SiO_2_ are very rare. Only a few studies reported the model establishment, prediction, and optimization for the mix proportions and compressive strength of PVA fiber-reinforced cementitious composites containing nano-SiO_2_. Besides, the mix proportion optimization of composite materials is generally determined experimentally, which resulted in a large amount of manpower and material resource consumption [38]. To reduce test consumption, improve test productiveness, and rapidly determine the best mix proportion of the composites, it is crucial to establish a suitable model to predict the compressive strength. In this study, the BP neural network will be used to propose a method for compressive strength prediction of PVA fiber-reinforced cementitious composites containing nano-SiO_2_. The BP neural network has been proven to exhibit a strong nonlinear mapping ability, and it can be extensively used in the construction and prediction of complex nonlinear models [39]. Besides, orthogonal test was conducted to establish a precise BP neural network, which can avoid the disadvantages of a neural network that cannot converge and fall into the local optimal solution and contains a certain reference value. Simultaneously, the genetic algorithm was applied to optimize the mix proportion of PVA fiber-reinforced cementitious composites containing nano-SiO_2_. The results of this study can effectively guide the mix proportion test of composite materials, reduce the human and material consumption, and improve the test efficiency.

## 2. Preliminary Processing and Analysis of Original Data

When executing a neural network, a certain number of training samples must be used; those used in this study were from Reference [40] and were processed as shown in Table 1 below. The mixtures 1–12 were prepared to study the influence of PAV fiber content on the compressive strength of cementitious composites. The mixtures 12–15 were prepared to study the influence of nanoparticle content on compressive strength of cementitious composites. Mixtures 15–18 were prepared to study the influence of quartz sand diameter on the compressive strength of cementitious composites. Mixture 19 was taken as the control mixture.

The concrete function of normalization is to induce the statistical distribution of unified samples. If the original data are used for analysis, the singular data in the sample will interfere with the test, which may increase the network training time or cause a convergence failure in the network. To avoid the phenomenon above and eliminate the calculation error caused by different data units and the system error caused by the difference in factor magnitude, the sample data shall be normalized [41] before further data analysis and processing, as follows:

(1) $$x\prime_{\left(m,i\right)}=\frac{x_{\left(m,i\right)}-x_{m,\min}}{x_{m,\max}-x_{m,\min}}$$

(2) $$y\prime_{pi}=\frac{y_{pi}-y_{p,\min}}{y_{p,\max}-y_{p,\min}}$$

where, $x_{\left(m,i\right)}$ is the content of component m in the mix proportion i; i is 1–19, m is 1–7, which corresponds to water, cement, quartz sand, fly ash, PVA fiber, nanoparticles, water reducing agent, respectively; $y_{pi}$ is the compressive strength corresponding to the mix proportion i; $y_{p,\min}$ is the minimum compressive strength of 19 composite specimens with different mix proportion; $y_{p,\max}$ is the maximum compressive strength of 19 composite specimens with different mix proportion; $y\prime_{pi}$ is the normalized compressive strength of composite i.

**Figure 1:**
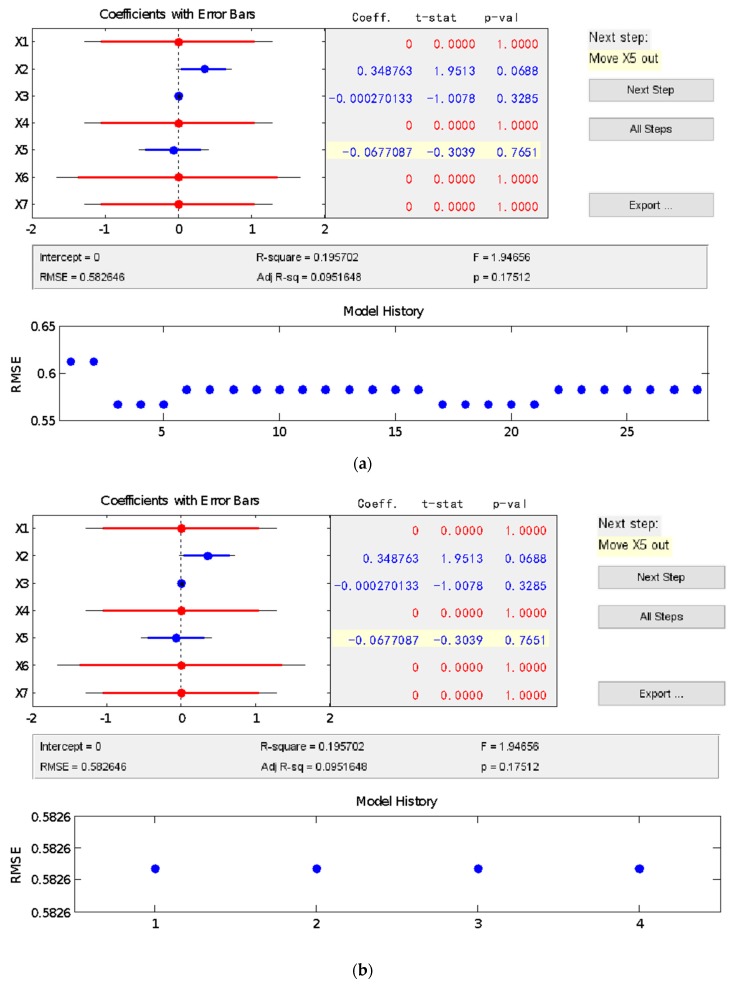

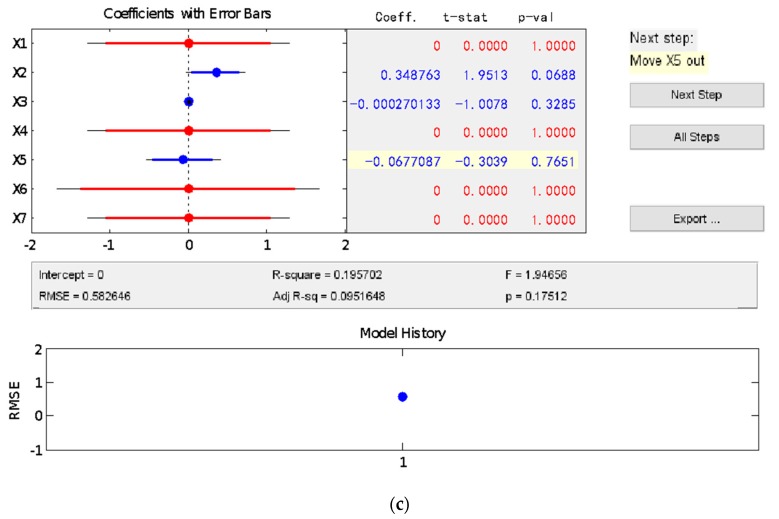
A multiple linear regression model constructed using the stepwise regression method. (**a**) First step, (**b**) Second step, (**c**) Third step.

According to the procedures shown in Figure 1, a multiple linear regression model [42] was built for the connection between the composite’s mix proportion and its compressive strength, based on the stepwise regression method, which was obtained as follows:

(3) $$y=0.348763x_{2}-0.000270133x_{3}-0.0677087x_{5}$$

where, $x_{2}$ is the normalized cement dosage; $x_{3}$ is the amount of quartz sand after normalization; $x_{5}$ is the amount of PVA fiber after normalization.

Utilizing the obtained linear regression model, the prediction results of the last four groups of data in Table 1 are presented in Table 2 below. From the prediction results, it can be perceived that the prediction results of the linear regression model for the compressive strength of PVA fiber-reinforced cementitious composites containing nano-SiO_2_ exhibits a large deviation.

Pearson correlation analysis [43] and variance analysis were performed to analyze the compressive strength of the composites obtained from the linear regression equation above and the actual compressive strength to assess the degree of interdependence between the two variables. Results from the Pearson correlation analysis are presented in Table 3, and the results of the variance analysis of the regression equation are presented in Table 4. As shown in Table 3, the conspicuousness is 0.652, which is a moderate correlation between 0.5 and 0.8 [44,45]. Therefore, according to the Pearson correlation analysis, the significance of this linear regression model is moderate. The variance analysis shown in Table 4 shows that the significance P > 0.05, i.e., when the error is 0.05 [46], no significant difference appears between the predicted and actual compressive strength values.

In general, when applying the linear regression method to predict the compressive strength of composite materials, many factors affect the compressive strength. Owing to the complex relationship between mix proportion and compressive strength, the linear regression method cannot reflect the relationship between mix proportion and compressive strength well enough to accurately predict the compressive strength of PVA fiber cementitious composites containing nano-SiO_2_.

To further prove the superiority of neural network, a quadratic multiple regression process is established as follows:

(4) $$y=1.1335x_{5}-0.0013x_{2}{}^{2}+0.0024x_{3}{}^{2}-0.0973x_{5}{}^{2}-0.1485x_{6}{}^{2}$$

where, $x_{2}$ is the normalized cement dosage; $x_{3}$ is the amount of quartz sand after normalization; $x_{5}$ is the amount of PVA fiber after normalization; $x_{6}$ is the amount of Nano-SiO_2_ after normalization.

As shown in Table 5 and Table 6, the fitting result of the quadratic nonlinear regression equation is better than that of linear equation, but the fitting effect is still general, and the prediction error is large, which is not suitable for prediction and optimization. Hence, the BP neural network was used to construct a nonlinear mapping relationship to predict the compressive strength of PVA fiber-reinforced cementitious composites.

## 3. Construction of Neural Network and Orthogonal Experiment

### 3.1. Construction of the Neural Network

The neural network algorithm simulates the working mode of human brain neurons by constructing the neuron structure [47] with a certain information transmission path and providing the connection weight function, thereby enabling artificial learning. Currently, it is widely [48] used to establish and predict complex nonlinear models. The BP feed-forward neural network was trained in accordance with the error backpropagation algorithm. Using the learning rule of the steepest descent method, the mathematical mapping relationship of the input–output mode is not required. In an arithmetic operation, the algorithm processes the signal forward propagation in the hidden layer; after comparing the output value with the actual value, the error backpropagation error returns to the hidden layer [49]. Finally, the feedback error returns to the input layer such that the neurons of each input layer share the error [50]. Simultaneously, the weights and thresholds are adjusted continuously until the output value of the output layer satisfies certain accuracy requirements. However, the BP neural network converges slowly and yields a local optimal solution, which leads to difficulty in obtaining the global optimal solution. To solve these problems, some new fast and effective algorithms were used as necessary. Among them, the Levenberg–Marquardt optimization algorithm exhibits fast convergence speed and good application performance [51], which is an optimal algorithm for medium-scale models. Therefore, according to the sample size and the sophisticated model studied herein, we selected the Levenberg–Marquardt algorithm to build the model.

The Levenberg–Marquardt algorithm applies the Jacobian and Hesse matrices to solve multidimensional optimization problems [52,53], whose principle is as follows:

(5) $$f\left(x\right)=0,\;x=\left[x_{0},x_{1},\cdots ,x_{n}\right]$$

The Jacobian matrix is as follows:

(6) $$J_{f}=\left[\begin{array}{ccc} \frac{\partial f_{1}}{\partial x_{0}} & \cdots & \frac{\partial f_{1}}{\partial x_{n}}\\ \vdots & \ddots & \vdots \\ \frac{\partial f_{n}}{\partial x_{0}} & \cdots & \frac{\partial f_{n}}{\partial x_{n}} \end{array}\right]$$

The Hessian matrix is as follows:

(7) $$H_{f}=\left[\begin{array}{cccc} \frac{\partial^{2}f}{\partial x_{0}{}^{2}} & \frac{\partial^{2}f}{\partial x_{0}\partial x_{1}} & \cdots & \frac{\partial^{2}f}{\partial x_{0}\partial x_{n}}\\ \frac{\partial^{2}f}{\partial x_{1}\partial x_{0}} & \frac{\partial^{2}f}{\partial x_{1}{}^{2}} & \cdots & \frac{\partial^{2}f}{\partial x_{1}\partial x_{n}}\\ \vdots & \vdots & \ddots & \vdots \\ \frac{\partial^{2}f}{\partial x_{n}\partial x_{0}} & \frac{\partial^{2}f}{\partial x_{n}\partial x_{1}} & \cdots & \frac{\partial^{2}f}{\partial x_{n}{}^{2}} \end{array}\right]$$

The basic form of the iterative equation is as follows:

(8) $$x^{x+1}=x^{s}+\Delta$$

(9) $$\Delta =-{\left(J_{f}^{T}J_{f}+\lambda I\right)}^{-1}J_{f}^{T}f_{\;}$$

where Δ is the neural network feedback weight (threshold) value change matrix; J is the Jacobian matrix, which is the first-order differential matrix of the training error to the threshold value; λ is the initial adjustment amount; I is the unit matrix; f is the training error matrix.

It is noteworthy that when λ is small, the Levenberg–Marquardt algorithm is similar to the Gauss–Newton algorithm. If the value is large, the Levenberg-Marquardt algorithm can be regarded as a gradient descent method, which is positively related to the error of the algorithm feedback process.

Based on the principle of momentum gradient descent, each layer of neurons in the BP neural network extracts a small batch of data for a small gradient descent through certain learning and training batches and obtains an exponential weighted average for a series of gradients to reduce the error and adjust the vibration, before gradually approaching the optimized value for model construction. The expression of the exponential weighted average is as follows:

(10) $$\lambda =\beta \times \lambda_{n-1}+\beta \times lrate\times \lambda_{n}$$

where $\lambda_{i}$ is the adjustment of the gradient i; β is the momentum factor, where (0,1) is used and the weight update is increased when two gradients are the same; otherwise, the update is reduced; lrate is the learning rate, which is positively related to the prominent weight change of the network in the iterative process.

**Figure 2:**
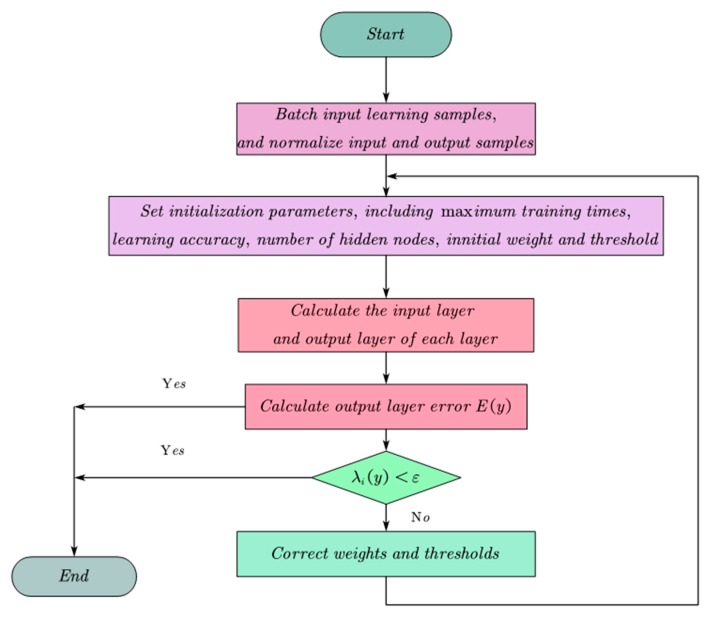
A flow chart of Levenberg–Marquardt algorithm.

In exponential weighting, each operation must obtain the average of an index and memorize it. In addition, each adjustment contains the information of all previous data. Figure 2 shows the flow chart of the Levenberg–Marquardt algorithm.

**Figure 3:**
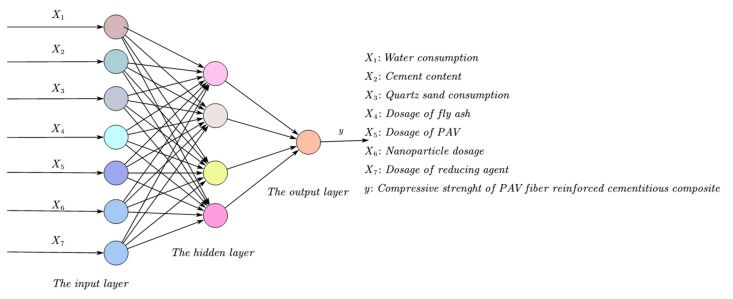
Architecture of three-layer backpropagation (BP) neural network model.

According to the principle of the Levenberg–Marquardt algorithm of the BP neural network, a three-layer neural network model was constructed, as shown in Figure 3. The compressive strength of the cementitious composite containing PVA fiber is primarily related to its mix proportion including water, cement, quartz sand, fly ash, PVA fiber, nano-SiO_2_ and water-reducing agent [54], correspondingly reflected by seven nodes in the input layer (seven components of an input vector). Additionally, one node in the output layer corresponds to the compressive strength. During training, three batches were divided by the raw data: 70% as training data, 15% as test data, and 15% as validation data, and model optimization is achieved by adjusting the relevant parameters and functions.

### 3.2. Initial Test of BP Neural Network

Prior to the neural network orthogonal test, a trial test was performed to preliminarily observe the matching of the BP neural network model to the PVA fiber-reinforced cementitious composite to prepare for the following orthogonal test for determining the specific parameters. The parameter settings of the initial trial test are presented in Table 7.

**Figure 4:**
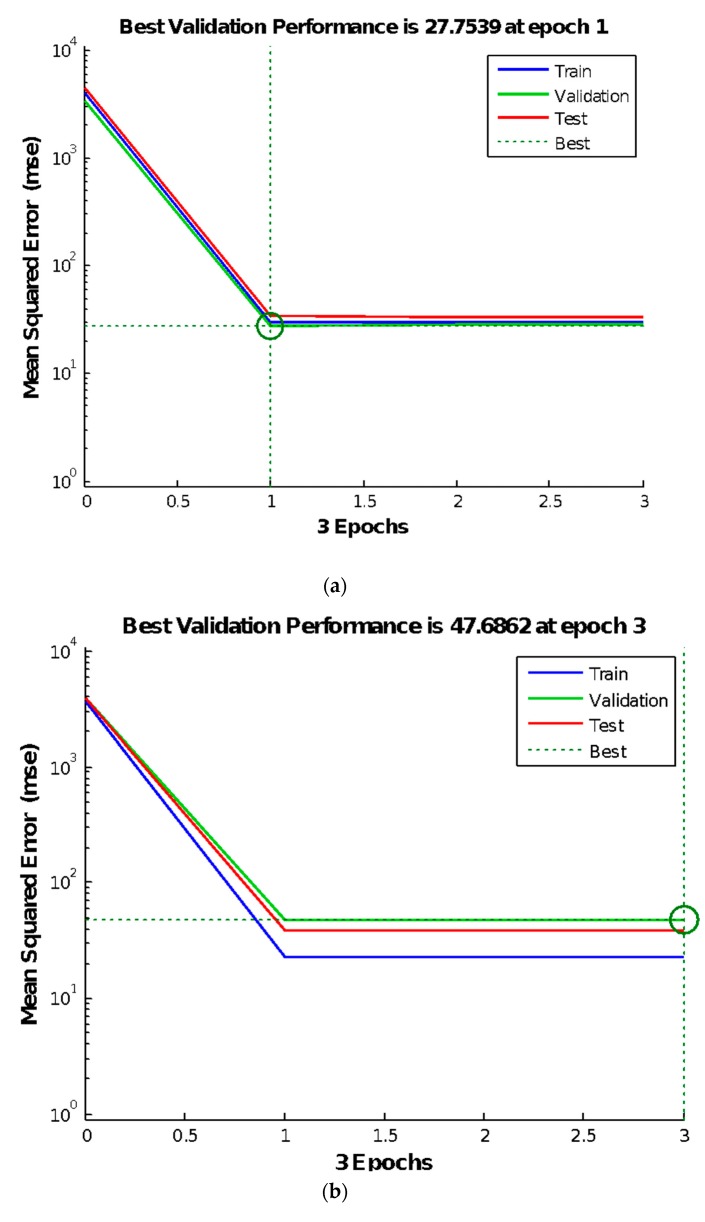

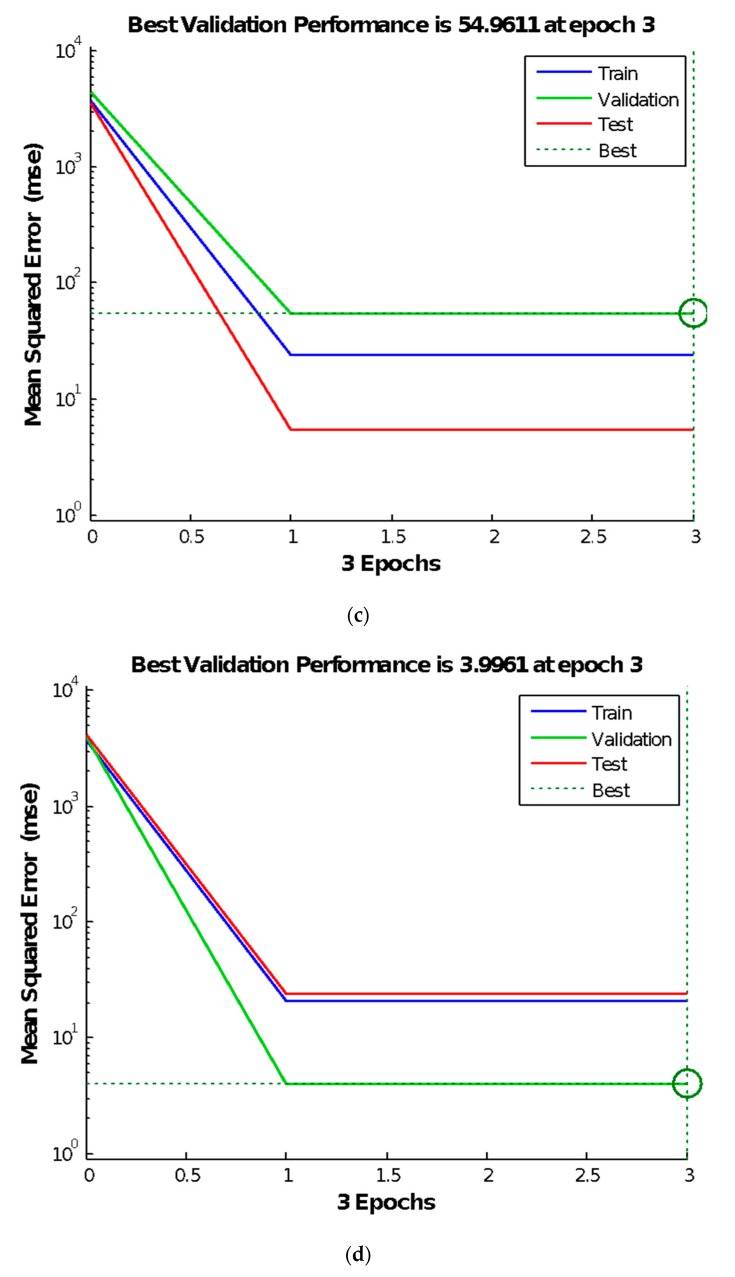
Results of the BP neural network trial test. (**a**) P-4c-k; (**b**) P-7c-k; (**c**) P-10c-k; (**d**) P-13c-k.

**Figure 5:**
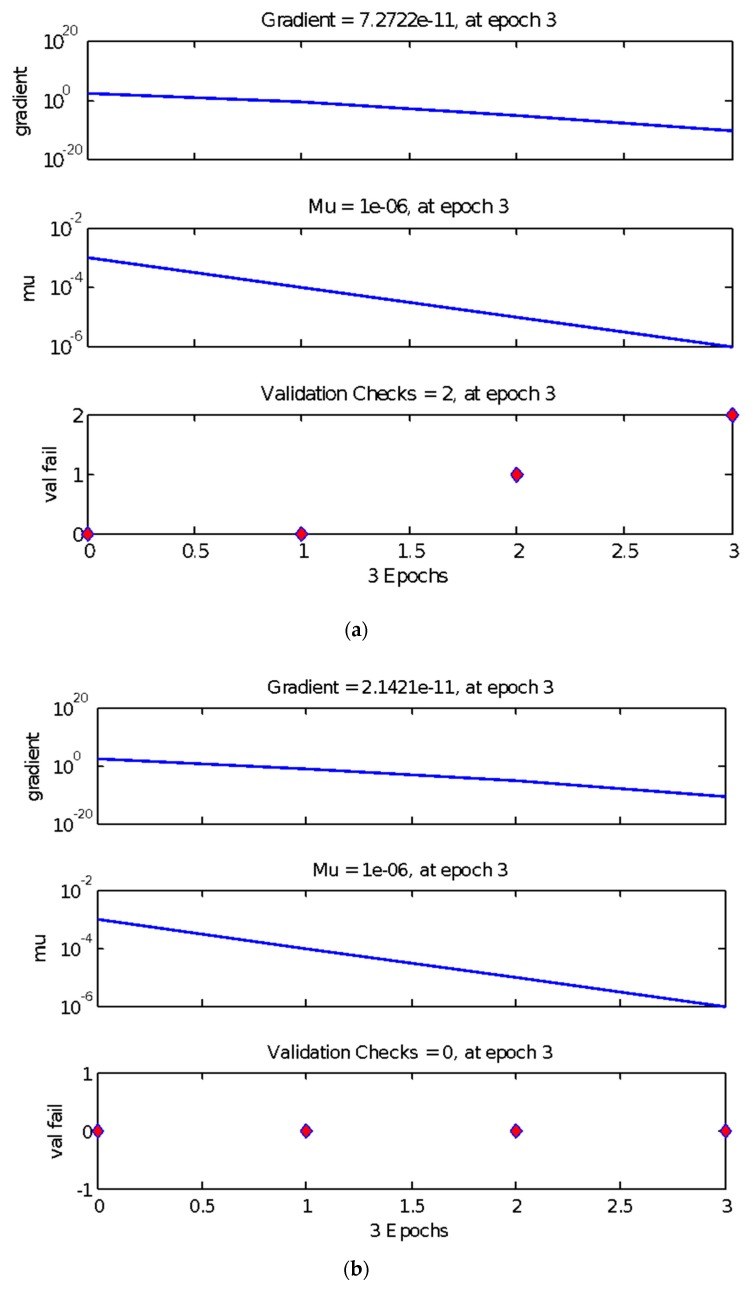

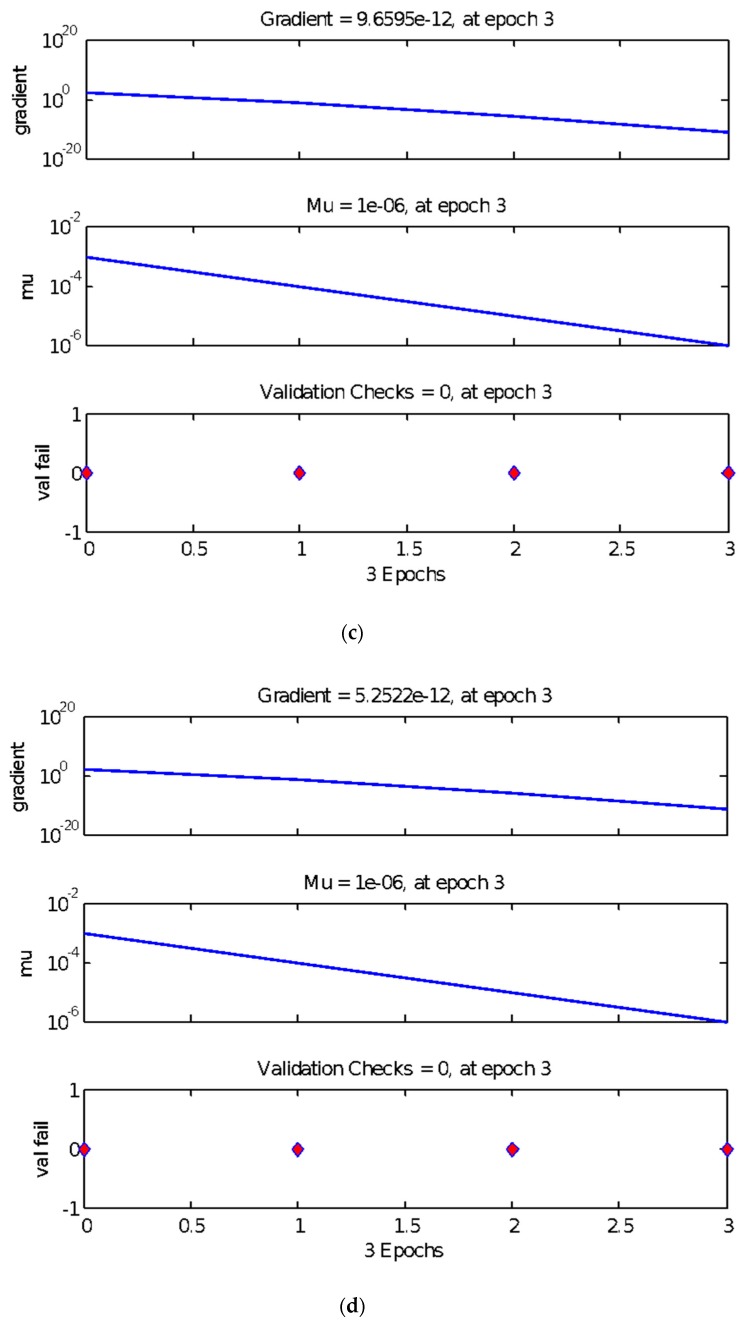
The relationship between gradient and learning times.(**a**) P-4c-k; (**b**) P-7c-k; (**c**) P-10c-k; (**d**) P-13c-k.

**Figure 6:**
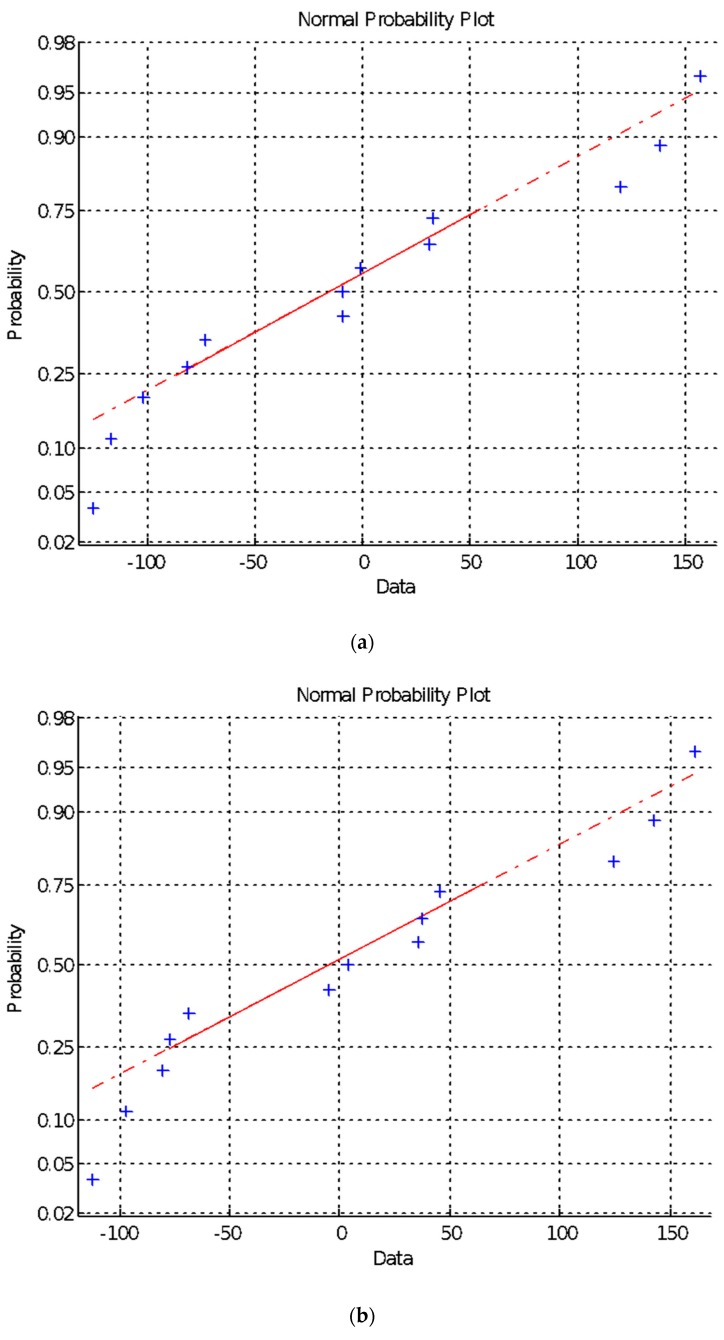

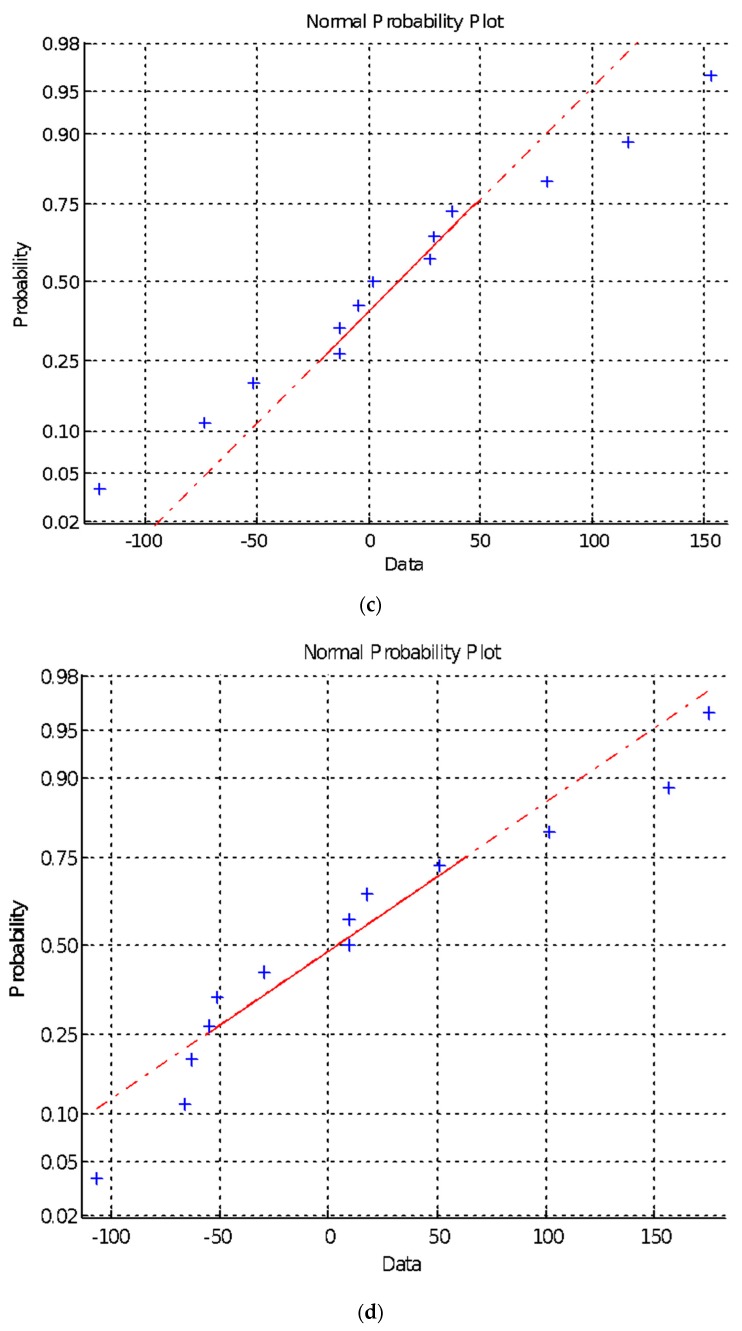
The relationship between gradient and mean square error of training data. (**a**) P-4c-k; (**b**) P-7c-k; (**c**) P-10c-k; (**d**) P-13c-k.

The results of the BP neural network trial test, the relationship between gradient and learning times, and gradient and mean square error of the training data are exhibited in Figure 4, Figure 5 and Figure 6, respectively, which are arranged from top to bottom and left to right according to the parameter level. As shown in these figures, after a certain number of iterations, the neural network can converge to obtain a relatively accurate solution. Therefore, the BP neural network was employed in the subsequent search. However, some disadvantages, such as obtaining the local optimal solution, remained [55]. Therefore, an orthogonal test must be performed to optimize the parameters of the neural network.

### 3.3. Orthogonal Test of Network Experiment

#### 3.3.1. Test Program

Through extensive experimental studies, it was discovered that when the Levenberg–Marquardt algorithm was used to establish the neural network model, the training effect was the best. Meanwhile, the hyperbolic tangent s-type (tansig) transmission function was utilized as the input layer transmission function, linear transmission function (purelin) as the output layer transmission function, and momentum gradient descent method (traingd) [56] as the reverse training function. The trial experiment indicated that in a specific training process, the number of hidden layer neurons, frequency of training, MSE, learning rate, momentum factor, and display interval times affected the performance of BP training.

To further optimize the model, the relevant parameters were selected more purposefully, the effects of interfering factors on the test results were avoided, and initial quantity interactions in the test were considered. In this study, an orthogonal test was performed to optimize the relevant parameters. During the trial, three experimental levels were designed for each parameter, whose values corresponding to the changes in each level are presented in Table 8 below, which were selected based on experience. Among them, the number of neurons was ascertained by the following empirical formula: $l=\sqrt{n+m}+a,\;a\in \left[1,10\right]$, where a is an integer obtained from the boundary of 1–10. The training times were determined according to the convergence times of the trial test. Furthermore, the remaining limit and median values within the corresponding allowable range were used such that the orthogonal test results were optimal. The predicted value of Group 19 obtained through the neural network model was recorded during the experiment.

During the test evaluation, the value of each factor at each level was compared with the predicted compressive strength value, the regression sum of the quadratic corresponding to each parameter value calculated, and regression analysis conducted while evaluating the significance of each factor and its interactions on the model. The initial parameters were adjusted until the model was optimized. In the experiment, Matlab was used to compile the algorithm, and the SPSS software was used for a single-factor ANOVA.

#### 3.3.2. Test Results and Analysis

To satisfy the requirements of concise expression, the number of hidden neurons, training times, mean square error, learning rate, momentum factor, and display interval times in the table above are represented by the characters A, M, N, B, C and D, respectively. A × B represents the interaction between the number of hidden neurons and learning rate by analogy, while A × C and B × C is similar to A × B. The orthogonal test header of the BP neural network is designed as shown in Table 9 [57,58].

The statistical results obtained from the experiment are exhibited in Table 10 [59], in which the interaction items do not directly affect the test results. The parameter values in a certain column correspond to the level of the orthogonal test in Table 8, which are distinguished by brackets when recording.

After a regression analysis of the experimental results, the parameters that affected the performance of the neural network the most can be obtained, while the total square sum of the sample regression is calculated as follows:

(11) $$T=\sum_{\beta =1}^{\alpha }\sum_{i=1}^{L_{evel}}\sum_{j=1}^{N_{um}}R_{ij},\;\bar{R}=\frac{T}{\alpha \times L_{evel}\times N_{um}}$$

(12) $$TT_{S}={\sum_{i=1}^{L_{evel}}\sum_{j=1}^{N_{um}}{\left(R_{ij}-\bar{R}\right)}^{2}}_{\;}$$

The sum of sample regression squares of a parameter change can be obtained as follows:

(13) $$TT_{A}=N_{um}{\sum_{i=1}^{L_{evel}}\left[k_{\beta }\left(i\right)-\bar{R}\right]}^{2}=\frac{{\sum_{i=1}^{L_{evel}}\left(\sum_{\chi =1}^{T_{otal}}R^{ij}\right)}^{2}}{N_{um}}-\frac{T^{2}}{L_{evel}\times N_{um}}$$

where $L_{evel}$ is the number of parameter layers; $N_{um}$ is the number of tests performed at a certain level; $R_{ij}$ is the test result of the first test conducted at level i; $T_{otal}$ is the total number of tests; $T_{otal}=L_{evel}\times N_{um}$; $TT_{A}$ is the square sum of variance of a parameter; $TT_{S}$ is the sum of squares of the total variance; α is the number of test parameters; β is the test parameter number; χ is the test number.

Meanwhile, each interaction item is regarded as an interfering factor, and the calculation method of the samples’ regression square sum is analogous to that of each single factor’s sum of sample regression square, whose ultimate arithmetic results are shown in Table 11.

By comparing the error with the sum of the samples’ regression squares caused by each influencing factor, it is clear that the mean square error and momentum factor have a negligible effect on the performance of the neural network; therefore, they are regarded as negligible accidental errors. The *F* test indicated that the greater the F value, the stronger was the sensitivity. According to the experimental results in Table 8, the sensitivities of A, M, A×B, A×C, and B×C to the experiment was highly significant. Nevertheless, the performance of the neural network was almost unaffected by N, B, C, and D. Therefore, according to the importance of the model, the sequence of factors from strong to weak was as follows: The interaction between number of hidden neurons and learning rate, number of hidden neurons, training times, interaction between training times and mean square error, interaction between number of hidden neurons and mean square error, number of display intervals, learning rate, mean square error, and momentum factor, among which the effect on the model was not significantly different when only the last four items were considered.

## 4. Model Parameter Optimization and Validation

### 4.1. Parameter Optimization

Based on the previous section, we can obtain the effect of each parameter change on the model and ultimately determine the best value of each parameter through range analysis, which can be determined in Table 12 and expressed as follows:

(14) $$\sum {}_{A}\left(i\right)=\sum_{\beta =1}^{N_{um}}R_{ij}$$

(15) $$k_{A}\left(i\right)=\frac{\sum_{A}\left(i\right)}{N_{um}}$$

(16) $$R_{A}=\underset{\gamma }{\max}k_{A}\left(i\right)-\underset{\gamma }{\min}k_{A}{\left(i\right)}_{\;}$$

where $N_{um}$ is the number of tests performed at a certain level; $R_{ij}$ is the result of the test conducted at level i; A is the influence factor; γ is the number of test parameters; and β is the test parameter number.

The results of range analysis reveal that the larger the range, the more significant is the parameter, which illustrates that the number of neurons in the hidden layer is the most remarkable. Other factors, including the learning rate, number of display intervals, and momentum factors affect the experiment results, whose degrees of effect are almost the same, which is consistent with the results of variance analysis.

According to the principle that the smaller the parameter value, the higher the utilization rate of the neural network and the better is the performance, when the parameter change does not significantly affect the model, the factor level value with the smaller performance index should be selected. Meanwhile, although the interval times, learning rate, mean square error, and momentum factor have little effect on the test results, their interactions impose definite effects on the test results. Therefore, the value should be selected such that its effect on the test results should be reduced to obtain more accurate test results and a higher degree of model fitting.

Combined with the analysis of range and variance, the training times and number of neurons in the hidden layer are significant when they function individually, whereas the number of neurons in the hidden layer, learning rate, and momentum factor are significant when they interact with each other. Meanwhile, the mean square error and number of display intervals are relatively small. Considering the operation performance of the neural network, the best parameter combination of the BP neural network is obtained: Training times 100 (Level 1), hidden layer neurons 2 (Level 1), mean square error 0.001 (level), learning rate 0.5006 (Level 2), display interval times 52 (Level 2), and momentum factor 0.503 (Level 2).

### 4.2. The Model Test

It was discovered that the predicted compressive strength was 59.2 MPa, which was closer to the real compressive strength of 58.2 MPa compared with any group of orthogonal experiments. Subsequently, we must examine the generalization ability of the constructed neural network, and then assess whether the network has been equated, in which an outstanding neural network is trained to forecast the last three groups of data in Table 1, whose results are shown in Table 13 below. It is clear that the error of the network’s simulation results can be controlled to within 11%, thereby avoiding over-fitting in training.

## 5. Results and Discussion

By applying the genetic algorithm in Matlab to optimize the mix proportion of the composite materials [60], two iterations of the genetic algorithm are performed in the main process [61]. One performs before the BP neural network to obtain individual population and fitness values for training the BP neural network. However, the other is performed after the BP neural network to obtain individuals that are brought into the BP neural network again to train the network, rendering the network more accurate. The individual fitness of the latter is obtained partly by the fitness function of the BP model and partly by the initial fitness function. The latter part is substituted into the BP neural network to render it more accurate. When the nonlinear mapping relationship established in the BP neural network is substituted as the fitness function, it can be inferred from a previous study that the fitness function can be calculated as follows [62]:

(17) $$f=\frac{1}{2}\times \max{\left(abs\left(t_{i}-purelin\left(w_{2}\times \tan sig\left(w_{1}\times m_{i}+b_{1}\right)+b_{2}\right)\right)\right)}^{2}$$

According to the Chinese Standard [63] and the literature [40], the water-binder ratio is 0.35–0.41; the water-cement ratio is 0.5–0.65; the cement-sand ratio is 1.22–1.36; the volume content of PVA fiber is 0–1.5%; the content of nano-SiO_2_ is 0–2.5%. The mathematical model is as follows [64]:

(18) $$\left\{ \begin{array}{l} \max f\left(x_{1},x_{2},x_{3},x_{4},x_{5},x_{6},x_{7}\right)\\ subject\left\{ \begin{array}{l} 350<x_{1}<410\\ 0.50<\frac{x_{1}}{x_{2}}<0.65\\ 1.22<\frac{x_{2}}{x_{3}}<1.36\\ 320<x_{4}<380\\ 0<x_{5}<13.65\\ 0<x_{6}<16.25\\ 1<x_{7}<5 \end{array}\right. \end{array}\right.$$

where $m_{i}$ is the input vector; $t_{i}$ is the target vector; $w_{1}$, $w_{2}$, $b_{1}$, $b_{2}$, are the input and output layer weight and threshold matrices, respectively; f is the nonlinear mapping constructed in the neural network model; $x_{1},x_{2},x_{3},x_{4},x_{5},x_{6},x_{7}$ are the mix proportions of the composite material.

To improve the efficiency of searching for the maximum, the chromosome code was written in real numbers, with a crossover probability of 0.6 and a mutation probability of 0.01 for executing the algorithm, while the number of single chromosome corresponded to the number of variables, which was 8 [60]. For each iteration through the algorithm, the current optimal individual was forced to participate in the next generation evolution. After 100 generations, the best mixture ratio was as follows: Water: cement: quartz sand: fly ash: PVA fiber: nano-SiO_2_: water reducer = 384:649:508:349:9.5:8.1:3.0. The BP neural network predicted the corresponding compressive strength, i.e., 68.7, which is superior to most of the compressive strengths in Table 1 (except for mixtures 13 and 16).

The differences from the best mix proportion in the literature [40] are shown in Table 14. As is shown in Table 14, the differences between the two optimal mix proportions are within 0.1%. According to the literature [40], the compressive strength of PVA fiber-reinforced cementitious composites containing nano-SiO_2_ can be enhanced when the content of PVA is 0–0.6%. Besides, with the increase of PVA fiber content, the compressive strength has no obvious increase or decrease trend. Therefore, the difference of PVA content can be ignored. Moreover, the compressive strength values of the two mix proportions are close, so the two mix proportions can be understood as the same mix proportion, which reflects the reliability of the BP neural network.

## 6. Conclusions

(1) The BP neural network model could reflect the complex nonlinear mapping relationship between the compressive strength and its mix proportion, which could facilitate in predicting the compressive strength of PVA fiber-reinforced cementitious composites. Moreover, using the genetic algorithm to optimize the BP neural network could effectively optimize the mix proportion of composite materials, whose results could be used in the composite mix proportion test and improve the test efficiency.

(2) The parameters of the BP neural network could be determined by an orthogonal test that considered the effect of the interaction among the parameters on the performance of the neural network for achieving a BP neural network model with good performance. The conspicuousness of each parameter for the performance of the BP neural network was from strong to weak in the following sequence: The interaction between the number of hidden neurons and learning rate, the number of hidden neurons, training times, the interaction between training times and mean square error, the interaction between number of hidden neurons and mean square error, the number of display intervals, the learning rate, the mean square error, and the momentum factor.

(3) The optimal mix proportion of the PVA fiber cementitious composite was water: cement: quartz sand: fly ash: PVA fiber: nano-SiO_2_: water reducer = 384:649:508:349:9.5:8.1:3.0, whose predicted compressive strength was 68.7 MPa. It was verified that the experimental results could be used to optimize the compressive strength of cementitious composites reinforced by PVA fibers.

## Figures and Tables

**Table 1:** Mix proportions of polyvinyl alcohol (PVA) fiber cementitious composites.

Mix No.	Water	Cement	Quartz Sand	Fly Ash	PVA Fiber	Nano-SiO_2_	Water Reducing Agent	Compressive Strength
	kg/m^3^	kg/m^3^	kg/m^3^	kg/m^3^	kg/m^3^	kg/m^3^	kg/m^3^	MPa
1	380	650	500	350	0	0	3	62.3
2	380	650	500	350	2.73	0	3	64.8
3	380	650	500	350	5.46	0	3	67.3
4	380	650	500	350	8.19	0	3	61.8
5	380	650	500	350	10.92	0	3	64.2
6	380	650	500	350	13.65	0	3	62.7
7	380	637	500	350	0	13	3	59.5
8	380	637	500	350	2.73	13	3	61.8
9	380	637	500	350	5.46	13	3	64.3
10	380	637	500	350	8.19	13	3	56.3
11	380	637	500	350	10.92	13	3	58.0
12	380	637	500	350	13.65	13	3	54.9
13	380	643.5	500	350	8.19	6.5	3	71.7
14	380	640.25	500	350	8.19	9.75	3	69.5
15	380	633.75	500	350	8.19	16.25	3	55.4
16	380	637	500	350	8.19	13	3	70.6
17	380	637	500	350	8.19	13	3	57.5
18	380	637	500	350	8.19	13	3	57.3
19	380	637	500	350	0	13	3	58.2

**Table 2:** The prediction results of the linear regression equation.

Mix No.	Compressive Strength	Predicted Compressive Strength	Relative Error
	MPa	MPa	%
16	70.6	143.2	102.9378
17	57.5	143.2	149.1723
18	57.3	143.2	150.0421
19	58.2	143.8	147.1125

**Table 3:** The prediction results of the linear regression equation.

Project	Correlation Coefficient	Saliency	Number of Cases
Y1	1.0	0.652	19
Y	1.0	0.652	19

**Table 4:** Variance analysis results of regression equation.

Project	Sum of Squares	Freedom	Mean Square	F	Saliency
Inter group combination	1.517	14	0.108	0.277	0.974
Weighting (between groups)	0.583	1	0.583	1.494	0.276
Variance (between groups)	0.933	13	0.072	0.184	0.994
In group	1.952	5	0.390	0	0

**Table 5:** Predicted results of the linear regression equation.

Mix no.	Compressive Strength	Predicted Compressive Strength	Relative Error
	MPa	MPa	%
16	70.6	50.2	0.2895
17	57.5	50.2	0.1276
18	57.3	50.2	0.1246
19	58.2	47.4	0.1855

**Table 6:** Predicted results of the linear regression equation.

Project	Correlation Coefficient	Saliency	Number of Cases
Y1	1.0	0.770	19
Y	1.0	0.770	19

**Table 7:** The initial parameter setting of the model.

No.	Number of Neurons in Saphenous layer	Training Times	Mean Square Error (MSE)	Learning Rate lrate	Momentum Factor β	Display Interval Times
P-4c-k	4	10000	0.0000001	0.005	0.1	10
P-7c-k	7	10000	0.0000001	0.007	0.5	19
P-10c-k	10	10000	0.0000001	0.01	0.9	25
P-13c-k	13	10000	0.0000001	0.1	1.2	35

**Table 8:** The corresponding parameter values of each test level.

No.	Number of Neurons in the Saphenous Layer	Training Times	Mean Square Error (MSE)	Learning Rate lrate	Momentum Factor β	Display Interval Times
Level 1	2	100	0.001	0.0010	0.0110	3
Level 2	16	500	0.00001	0.5006	0.5030	52
Level 3	32	1000	0.0000001	1.0001	0.1000	101

**Table 9:** The head of the orthogonal test considering the interaction.

Level	A	M	N	B	(A _×_ B)_1_	(A _×_ B)_2_	C	(A _×_ C)_1_	(A _×_ C)_2_	(B _×_ C)_1_	D	(B _×_ C)_2_
Column number	1	2	3	4	5	6	7	8	9	10	11	13

**Table 10:** The sample deviation analysis results of each interaction test.

NO.	A	M	N	B	A _×_ B		C	A _×_ C		B _×_ C	D	B _×_ C	R
	1	2	3	4	5	6	7	8	9	10	11	12	
1	2	100	0.001	0.0010	(1)	(1)	0.0110	(1)	(1)	(1)	3	(1)	64.4
2	2	500	0.00001	0.5006	(1)	(1)	0.5030	(2)	(2)	(2)	52	(2)	60.3
3	2	1000	0.0000001	1.0001	(1)	(1)	0.1000	(3)	(3)	(3)	101	(3)	62.9
4	2	100	0.001	0.0010	(2)	(2)	0.0110	(1)	(1)	(1)	3	(1)	59.9
5	2	500	0.00001	0.5006	(2)	(2)	0.5030	(2)	(2)	(2)	52	(2)	60.9
6	2	1000	0.0000001	1.0001	(2)	(2)	0.1000	(3)	(3)	(3)	101	(3)	63.0
7	2	100	0.001	0.0010	(3)	(3)	0.0110	(1)	(1)	(1)	3	(1)	62.9
8	2	500	0.00001	0.5006	(3)	(3)	0.5030	(2)	(2)	(2)	52	(2)	63.6
9	2	1000	0.0000001	1.0001	(3)	(3)	0.1000	(3)	(3)	(3)	101	(3)	64.7
10	16	100	0.001	0.0010	(2)	(2)	0.0110	(1)	(1)	(1)	3	(1)	62.0
11	16	500	0.00001	0.5006	(2)	(2)	0.5030	(2)	(2)	(2)	52	(2)	60.6
12	16	1000	0.0000001	1.0001	(2)	(2)	0.1000	(3)	(3)	(3)	101	(3)	61.2
13	16	100	0.001	0.0010	(3)	(3)	0.0110	(1)	(1)	(1)	3	(1)	62.5
14	16	500	0.00001	0.5006	(3)	(3)	0.5030	(2)	(2)	(2)	52	(2)	64.1
15	16	1000	0.0000001	1.0001	(3)	(3)	0.1000	(3)	(3)	(3)	101	(3)	61.1
16	16	100	0.001	0.0010	(1)	(1)	0.0110	(1)	(1)	(1)	3	(1)	60.7
17	16	500	0.00001	0.5006	(1)	(1)	0.5030	(2)	(2)	(2)	52	(2)	61.1
18	16	1000	0.0000001	1.0001	(1)	(1)	0.1000	(3)	(3)	(3)	101	(3)	63.7
19	32	100	0.001	0.0010	(3)	(3)	0.0110	(1)	(1)	(1)	3	(1)	61.7
19	32	500	0.00001	0.5006	(3)	(3)	0.5030	(2)	(2)	(2)	52	(2)	60.5
21	32	1000	0.0000001	1.0001	(3)	(3)	0.1000	(3)	(3)	(3)	101	(3)	60.3
22	32	100	0.001	0.0010	(1)	(1)	0.0110	(1)	(1)	(1)	3	(1)	62.4
23	32	500	0.00001	0.5006	(1)	(1)	0.5030	(2)	(2)	(2)	52	(2)	61.5
24	32	1000	0.0000001	1.0001	(1)	(1)	0.1000	(3)	(3)	(3)	101	(3)	62.8
25	32	100	0.001	0.0010	(2)	(2)	0.0110	(1)	(1)	(1)	3	(1)	61.8
26	32	500	0.00001	0.5006	(2)	(2)	0.5030	(2)	(2)	(2)	52	(2)	64.5
27	32	1000	0.0000001	1.0001	(2)	(2)	0.1000	(3)	(3)	(3)	101	(3)	62.9

**Table 11:** Results of sample regression variance analysis.

Interfering Factor	Sum of Squares of Sample Regression $TT_{A}$	Freedom f	Sum of Mean Regression Squares MS	F	Saliency
A	2.060	2	1.030	0.391	0.683
M	0.828	1	0.828	0.314	0.582
N	0	0	0	0	0
B	0	0	0	0	0
A×B	0.112	1	0.112	0.043	0.839
C	0.000	0	0	0	0
A×C	1.023	1	1.023	0.388	0.542
B×C	1.023	1	1.023	0.388	0.542
D	0	0	0	0	0
e(N,B,C,D)	0	0	0	0	0

**Table 12:** The results of the range analysis.

Project	A	M	N	B	C	D
F1	62.54277	62.04506	62.04506	62.04506	62.04506	62.04506
F2	62.18327	61.91194	61.91194	61.91194	61.91194	61.91194
F3	62.03376	62.52182	62.52182	62.52182	62.52182	62.52182
Range R	3.426	2.284	2.284	2.284	2.284	2.284

**Table 13:** The results of the BP neural network generalization ability test.

Mix No.	Compressive Strength	Predicted Compressive Strength	Relative Error
	MPa	MPa	%
16	70.6	63.2	10.522
17	57.5	60.8	5.7005
18	57.3	58.9	2.7342
19	58.2	59.2	1.7765

**Table 14:** Optimum mix of PVA fiber-reinforced cementitious composites containing nano-SiO_2._

Source	Water-Cement Ratio	Cement-Sand Ratio	Volume Content of PVA Fiber	Content of Nano-SiO_2_	Compressive Strength
	%	%	%	%	MPa
Prediction model	0.59	1.28	1.0	0.9	68.7
Literature [40]	0.60	1.29	0.9	1.0	71.7
